# The Energy Potential, Environmental Impact, and Occupational Health and Safety Potential of Biogas Obtained from Filter Cake in Artisanal Panela Production

**DOI:** 10.3390/bioengineering13020182

**Published:** 2026-02-04

**Authors:** Reni Danilo Vinocunga-Pillajo, Estela Guardado Yordi, Josselyn Pico Poma, Leidy Pico Poma, Diego Sarabia Guevara, Karel Diéguez-Santana, Amaury Pérez Martínez

**Affiliations:** 1 Facultad de Ciencias de la Vida, Universidad Estatal Amazónica, Puyo 160101, Pastaza, Ecuador; e.guardadoy@uea.edu.ec (E.G.Y.); jp.picop@uea.edu.ec (J.P.P.); da.sarabiag@uea.edu.ec (D.S.G.); 2 Ministerio de Educación—Zonal 3, Distrito 16D01, Puyo 160101, Pastaza, Ecuador; leidydf6@gmail.com; 3 Biomolecules Discovery Group, Universidad Regional Amazónica IKIAM, Km 7 Vía Muyuna, Tena 150101, Napo, Ecuador; karel.dieguez@ikiam.edu.ec

**Keywords:** biogas, cachaza, calorific value, mathematical models, panela, simulation, stoichiometry

## Abstract

Filter cake (or cachaza), a residue generated in the artisanal production of panela, represents an under-explored source of renewable energy in the Ecuadorian Amazon. Valorizing filter cake could reduce the use of solid biomass and emissions associated with traditional combustion. Our objective was to determine the energy potential of the biogas obtained and its contribution to the sustainability of the panela (unrefined cane sugar) production system. A sequential procedure was applied that included the physicochemical characterization of filter cake, feed flow modeling, and stoichiometric simulation under mesophilic conditions. The anaerobic digestion of filter cake with the optimal Composition 6 generated up to 1736.40 m^3^·day^−1^ of biogas with 40.7% methane and a calorific value of 14,350 kJ·m^−3^. This was enough to replace 1.24 t·day^−1^ of wood or 2.38 t·day^−1^ of bagasse in the production system. This represents an annual saving of 631.08 t of solid biomass, equivalent to conserving 3.63 ha·year^−1^ of the Amazon rainforest. The Tool for the Reduction and Assessment of Chemical and Other Environmental Impacts (TRACI) analysis showed impacts on climate change (17.40 kg CO_2_ eq/m^3^) and acidification (0.00516 kg SO_2_ eq/m^3^), attributable to unburned methane and residual H_2_S. Meanwhile, the social assessment using the Occupational Health and Safety Potential (OHSP) indicator showed high risks in terms of handling filter cake and cleaning the digestate.

## 1. Introduction

Globally, around 80% of Saccharum officinarum crops are used for sugar production and 20% for ethanol [1]. In 2020, around 1869.7 million tons of sugarcane were harvested, and Brazil was the main producer, growing 757.1 million tons, followed by India, China, Pakistan, and Thailand [2]. Brazilian productivity can achieve up to 72 t/ha, with the southeast region accounting for 60% of national production [3]. The sugar industry generates approximately 279 million metric tons of waste each year, including bagasse and filter cake [4]. The latter, also known as cachaza, is produced during juice clarification and has a high moisture content (≈75%) as well as compounds such as cellulose, hemicellulose, lignin, sucrose, and minerals [5]. The composition of filter cake varies depending on the type of sugarcane, the soil, the climate, and the efficiency of the milling process [6].

The province of Pastaza, in the Ecuadorian Amazon, has an estimated sugarcane production potential of 4500 hectares, with an average yield of 55 t/ha, mainly for the production of derivatives such as panela in blocks, granulated panela, aguardiente (a type of sugarcane liquor), and fresh sugarcane sticks [7]. The local sugarcane industry is made up of more than 110 small factories, which, alongside their products, generate a considerable amount of organic waste that accumulates around these facilities [8]. This activity depends on the use of solid biomass wood and bagasse as a source of thermal energy, which leads to a high consumption of forest resources and the emission of incomplete combustion gases. Therefore, the valorization of filter cake as an energy substrate is a strategic alternative to reduce deforestation, decrease the use of traditional biomass, and strengthen the sustainability of artisanal sugarcane systems. However, there is no research available that integrates stoichiometric simulation with TRACI (Tool for the Reduction and Assessment of Chemical and Other Environmental Impacts) and social life cycle assessment (S-LCA) tools to demonstrate the energy potential of biogas obtained from filter cake and its environmental and social benefits in artisanal panel systems.

The processing of sugarcane using rudimentary technologies continues to be common practice within small factories in the province of Pastaza. In several sugar mills (paneleras), filter cake is considered a waste product due to its high organic load, with chemical oxygen demand (COD) values of up to ≈90,000 mg/L and biochemical oxygen demand (BOD_5_) values of up to ≈40,000 mg/L [9]. The final disposal of filter cake is usually carried out without prior treatment, thus intensifying the contamination of soils, water bodies, and areas surrounding the trapiches [10]. Ref. [11] reported that 71.4% of sugar mills do not apply any kind of environmental management, while ref. [12] identified that 89.5% of environmental impacts come from processes related to the management of waste, such as filter cake, and other stages of the panela production process and cane cutting.

Anaerobic digestion represents an appropriate technological option for the treatment of agro-industrial waste with high moisture content, such as filter cake. This technology, in addition to being economically accessible, allows for the production of biogas as a renewable energy source and a stabilized by-product (digestate) with potential use as fertilizer [13]. Previous records of sugarcane waste digestion have focused on kinetic estimates or laboratory conditions, without incorporating a comprehensive approach that links biogas production with environmental sustainability indicators. This gap limits the possibility of comparing the energy efficiency of biogas with traditional fuels and makes it difficult to quantify its real environmental benefits.

The yield of anaerobic digestion is directly dependent upon the type of substrate used [14]. One study found that the combination of filter cake with agricultural waste achieved a production of 120 m^3^/t of methane (CH_4_), whereas the exclusive use of filter cake yielded outputs ranging from 73.7 to 129.5 mL/g of CH_4_ [15]. Similarly, ref. [16] reported the generation of 0.360 m^3^ of biogas from 10 kg of cattle manure, and ref. [17] achieved an 81% biogas yield using vinasses as substrate. The quality of biogas is associated with the proportion of methane present, which is the component with the highest calorific value. In simple applications, such as stoves, heaters, or generators, the removal of carbon dioxide (CO_2_) is not required. However, for more demanding uses like vehicle fuel, it is necessary to purify the biogas to maximize its energy efficiency [18].

In addition, the valorization of organic residues within local circular bioeconomy schemes supports decentralized energy generation and reduce environmental burdens associated with waste accumulation. Studies on coastal biomass management have demonstrated that organic residues possess technical and economic potential for bioenergy generation, including biogas production, soil improvement products and biochar, when adequate collection and processing technologies are implemented [19]. This perspective supports the relevance of evaluating filter cake as a locally available substrate for renewable energy recovery.

This study proposes a comprehensive approach applied to artisanal panela production, combining stoichiometric simulation in CHEMCAD [20,21] with the TRACI and S-LCA tools. This integration enables a simultaneous analysis of the energy yield of biogas obtained from filter cake and the environmental impacts associated with its use. It also incorporates the social dimension linked to the operation of the biodigester, offering a more complete view of the production system. In this way, a technical basis is established that contributes to optimizing the use of agro-industrial waste and promoting sustainable practices in artisanal panela systems.

The analysis of environmental impact using the TRACI tool allows for the evaluation of emissions associated with thermochemical and biological processes by considering compounds such as CH_4_, CO_2_, H_2_S, and NO_x_ [22]. This methodology classifies impacts into categories such as climate change, soil acidification, tropospheric ozone formation, eutrophication, human toxicity, photochemical smog, and ozone depletion [23], providing a quantitative basis for comparing energy alternatives to the use of fossil fuels or solid biomass. The choice of TRACI 2.2 over methodologies such as life cycle assessment (LCA), GREENSCOPE (Gauging Reaction Effectiveness for the Environmental Sustainability of Chemistries with a Multi-Objective Process Evaluator), or WAR (Waste Reduction Algorithm) is justified by its ability to estimate environmental impacts directly from simulated process flows, without requiring complete life cycle inventories. It sets itself apart from LCA, which requires a database, or GREENSCOPE and WAR, which focus on the efficiency and safety of large-scale chemical plants.

In addition to the environmental component, the sustainability of the sugarcane industry includes social and occupational dimensions. In this study, S-LCA was considered to identify occupational health and safety risks for personnel involved in the operation of the biodigester. The Occupational Health and Safety Potential (OHSP) indicator allowed for the quantification of hours potentially lost due to workplace accidents. Therefore, this study methodologically integrated CHEMCAD simulation, S-LCA, and the TRACI model and applied it to the case of an artisanal sugarcane mill in Pastaza. This enabled the simultaneous estimation of biogas yield, solid biomass savings, and associated environmental impact categories, providing a technical basis for the energy transition of the Ecuadorian sugarcane sector.

Based on the above, this study aimed to determine the energy potential, environmental impact, and OHSP of biogas obtained from the bagasse generated in the artisanal production of panela in Pastaza, using simulations in CHEMCAD v6.3.1 (Datacor, Inc., Pittsburgh, PA, USA) software, the TRACI v2.2 tool (EPA, USA), and the S-LCA OHSP indicator to estimate the sustainable performance of the panela system.

## 2. Materials and Methods

**Figure 1:**
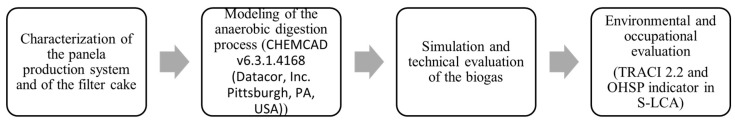
Heuristic diagram illustrating the sequential workflow applied in the study, from system characterization and process modeling to simulation-based performance evaluation and integrated environmental and occupational assessment.

This research followed a sequential methodological procedure aimed at assessing the energy use of filter cake and its effect on the panela system. The first stage involved collecting primary and secondary information from the plant in Pastaza, including data on milling capacity, filter cake generation, fuel consumption, and operating conditions. Based on this, the filter cake was characterized, and representative compositions of moisture, organic matter, and ash were defined, which served as direct input for modeling anaerobic digestion and defining input and output streams in the diagram of the process (Figure 1).

The final phase integrated modeling and simulation of the process in CHEMCAD with two levels of evaluation. First, the calorific value of the biogas and the amount of wood and bagasse that can be replaced in the boiler were calculated, consistent with actual plant data. Second, TRACI 2.2 was applied together with the OHSP indicator within the S-LCA approach to estimate environmental impacts and occupational risks.

### 2.1. Data Collection

The information used to characterize the filter cake was obtained from scientific sources indexed in international databases (Scopus, SciELO, Elsevier, Springer, and Wiley), using keywords such as filter cake, biogas, and anaerobic digestion. Recent (≤5 years) average compositions were prioritized, including moisture, ash, fiber, protein, lipids, and sugars. The information collected was compared with local data on sugarcane yield and panela production in the province of Pastaza [7,24]. This allowed the average compositions obtained from the literature to be adjusted, and it ensured their representativeness in the Amazonian context.

### 2.2. Characterization of the Filter Cake

The filter cake composition was analyzed based on information obtained from recent scientific literature, prioritizing experimental reference values. Data on crude protein, lipids, ash, sucrose, total fiber, and their fractions were incorporated, along with profiles of amino acids, fatty acids, minerals, and bioactive compounds reported by [5,25,26,27].

### 2.3. Modeling of the Process

Different boundary conditions for anaerobic digestion were analyzed by considering the upper and lower limits of the main components of the filter cake (Table 1). The purpose was to establish a representative range of compositions, including a base profile, in order to evaluate the system’s response to possible variations in the raw material.

Simulation scenarios were defined based on different feed flow levels (Table 2) by adjusting the percentage of available bagasse according to the theoretical value for the province of Pastaza. These combinations allow simulations to be run that show the behavior of the system under variable load conditions, with an emphasis on the amount of biogas in terms of CH_4_, CO_2_, and H_2_S.

### 2.4. Simulation of the Process

The simulation was performed in CHEMCAD v6.3.1 (Datacor, Inc., Pittsburgh, PA, USA) by modeling the anaerobic digestion of filter cake using mass balances. Daily capacity was estimated based on sugarcane data in Pastaza [7] and by considering usable area and land set aside for panela. The operating conditions were a mesophilic temperature of 25–35 °C and pH of 6.8–7.2 [28]. These parameters ensure microbiological stability in anaerobic processes.

The simulation was developed under a theoretical scenario assuming complete stoichiometric conversion of the organic compounds defined in the reaction set. This condition allows exploration of the maximum technical potential of the system for comparative purposes. Under real operating conditions, biological limitations, partial inhibition effects, microbial kinetics, and unavoidable operational losses may reduce the effective conversion, leading to lower methane yields than those predicted by the model. Therefore, simulated values should be interpreted as reference estimates rather than direct experimental performance.

#### Estimation of Filter Cake Production in Pastaza

The estimate was made according to the availability of sugarcane and the percentage of filter cake extracted. Sugarcane production and the percentage of usable sugarcane crops were established according to [7], while the estimated cane yield and percentage used for panela production were set according to [29], and the percentage of filter cake was determined as proposed by [30]. Based on these parameters and considering an annual operating period of 365 days, a tentative daily filter cake production of 9760 kg d^−1^ was estimated, and this value was used as a reference for defining feed flows and process simulation scenarios.

### 2.5. Technical Evaluation of Biogas

The Low Heating Value (LHV) was obtained by extrapolating the values published in [31]. The LHV of wood (*Eucalyptus globulus*) was obtained from [32] and that of bagasse from Equation (1) proposed by [33].

LHV = [(18,260 − 207.63 (moisture% sample) − 182.6 (ash% sample) − 31.14 (brix% sample)] kJ·kg^−1^ (1)

The energy balance equation $Q_{biogas}=Q_{wood}$ and $Q_{biogas}=Q_{bagasse}$ was used to estimate the amount of wood and bagasse that can be replaced by the biogas generated, considering the heating values of the three fuels evaluated.

The energy savings obtained by replacing solid biomass with biogas were compared with the average aboveground biomass of the Amazon rainforest to establish an ecological equivalence. To do this, a value of 174 Mg ha^−1^ was used as the average dry biomass density reported by [34], and an average individual biomass of 0.70 t tree^−1^. Using these references, the proportion of wood replaced that would correspond to the preserved forest area and number of equivalent trees was calculated, thus linking the use of biogas to the potential for rainforest preservation in the Amazon.

The results were compared with recent studies on the anaerobic digestion of filter cake and sugarcane residues carried out by [15,35,36]. The reference datasets included the organic matter composition, moisture content, and lignin content of filter cake, along with the experimental biogas and methane yields reported in those studies. The comparison covered total biogas volume, methane (CH_4_) fraction, and hydrogen sulfide (H_2_S) concentration, and there was a general alignment between local results and international experimental ranges.

### 2.6. Environmental Assessment with TRACI 2.2

In estimating the environmental impact using TRACI 2.2 (EPA, USA), only the gaseous flows generated during anaerobic digestion were considered, as they constitute the main source of direct emissions associated with the energy use of biogas. Liquid and solid flows—corresponding to digestate and process sediments—were not included because their impact requires further treatment depending on the type of disposal or agricultural use, which exceeds the system boundaries defined for this assessment. The quantitative analysis focused on emissions of CH_4_, CO_2_, H_2_S, and NO_x_, selected for their significant contribution to the categories of the impact of climate change (kg CO_2_ eq), acidification (kg SO_2_ eq), and tropospheric ozone formation (kg O_3_ eq), in accordance with the methodological guidelines of the TRACI 2.2 model.

The substances considered for each category were CH_4_ and CO_2_ for climate change, NO_x_ and H_2_S for acidification, and CH_4_ and NO_x_ for photochemical smog. The composition of the biogas was obtained from experimental results. For natural gas (≈98% CH_4_), liquefied petroleum gas (LPG), and propane, estimated emission values for complete combustion were used based on technical data [37,38,39]. The impact calculation consisted of multiplying the estimated mass of each substance by its corresponding TRACI characterization factor.

### 2.7. Occupational Health and Safety Potential (OHSP)

The study incorporated the occupational health and safety component through the use of the OHSP indicator, developed by [40], as a midpoint indicator within the S-LCA. This facilitated the quantification of hours potentially lost due to occupational accidents in each phase of the biogas production process (Equation (2)). The reference values came from the [41] database in conjunction with the values from the study panel. The mathematical model is described by the following relationship:

(2) $$OHSP_{n,a}=CF_{OHS,n,a}\times WH_{n}$$

where

$CF_{OHS,n,a}$ is the occupational characterization factor, calculated as:

(3) $$CF_{OHS,n,a}=\frac{24\times (\mathrm{Accidents}\;\mathrm{resulting}\;\mathrm{in}\;\mathrm{lost}\;\mathrm{days})}{\mathrm{Hours}\;\mathrm{worked}\;\mathrm{per}\;\mathrm{year}}$$

$WH_{n}$ corresponds to the total work hours in stage n of the production process.

The OHSP indicators module reports that the agro-industrial sector has a global average rate of 2.5 accidents resulting in lost days per 100 workers, with an average annual working time of 2000 h per employee and an average duration of 4 days lost per accident. These values were applied as reference parameters, adjusted proportionally to the actual hours of the simulated process.

The filter cake-based biogas system was divided into four operational stages: receiving and preparing the filter cake, loading and feeding the biodigester, operating and controlling anaerobic digestion, and cleaning and discharging the digestate. At each stage, the main exposure factors were identified and the OHSP indicator was estimated, taking into account the characteristics of the work, the annual duration, and the nature of the existing risks.

## 3. Results

### 3.1. Bibliographic Characterization

Table 3 shows the compounds present in filter cake, including macronutrients, essential and non-essential amino acids, saturated and unsaturated fatty acids, minerals, and phytochemicals with functional potential. This characterization allows for the identification of components suitable for agro-industrial use, such as structural fiber, phytosterols, and residual sugars.

The stoichiometric reactions presented in Table 4 represent simplified global conversion pathways used to approximate the overall transformation of carbohydrates, proteins and lipids into methane and carbon dioxide under idealized steady-state conditions. This formulation assumes complete conversion and does not explicitly account for intermediate metabolites, microbial biomass growth or kinetic limitations, which are addressed in more detailed anaerobic digestion models such as Anaerobic Digestion Model No. 1 (ADM1) [42]. Therefore, the reactions were selected to estimate comparative and upper-bound technical potential rather than predictive operational performance.

### 3.2. Mathematical Modeling

Mathematical models for predicting biogas production were developed based on the general mass balance equation and the chemical composition of the waste. The modeling considered the main organic fractions present in filter cake, allowing the formulation of stoichiometric reactions that describe the conversion of organic matter during anaerobic digestion (Table 4).

### 3.3. Modeling of the Process

#### 3.3.1. Simulation of the Anaerobic Digestion Process

**Figure 2:**
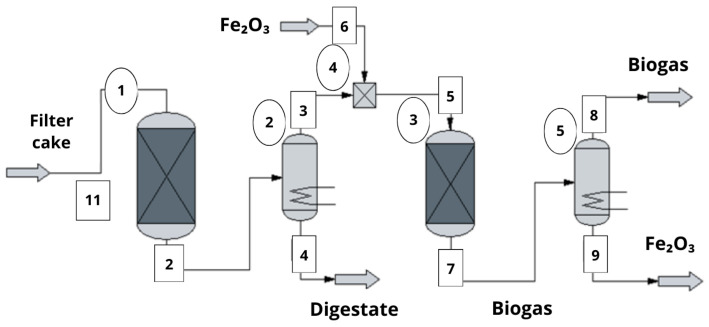
Technological diagram concerning biogas production.

The simulated process starts with the storage of filter cake (Unit 1 in Figure 2), which is subsequently fed into the anaerobic digester operated under mesophilic conditions at approximately 25 °C. This operating temperature was selected to maintain stable microbial activity during anaerobic digestion [28]. After digestion, two main output streams are generated: digestate and biogas. The digestate is discharged through Stream 4, while the biogas is conveyed through Stream 5 toward a purification unit.

In the purification stage, the biogas comes into contact with iron (III) oxide supplied as nanoparticles or granular hematite. This interaction promotes the removal of hydrogen sulfide from the gas phase. The solid products formed during this stage are discharged through Stream 9, while the purified biogas exits the system as the final gaseous product (Figure 2).

#### 3.3.2. Estimation of Bagasse Production in the Sugar Mill

The estimated amount of bagasse generated in the province of Pastaza is approximately 9760 kg·d^−1^, considering an extraction rate of 4% per ton of processed sugarcane [29]. Based on a processing capacity of 10 t·d^−1^, a total of 400 kg·d^−1^ of filter cake was estimated as available substrate for biogas production in the simulation. To evaluate system performance under variable operating conditions, the simulation was performed using different feedstock capacities (Table 5).

### 3.4. Process Simulation

#### 3.4.1. Base Production Case According to the Literature Review

**Figure 3:**
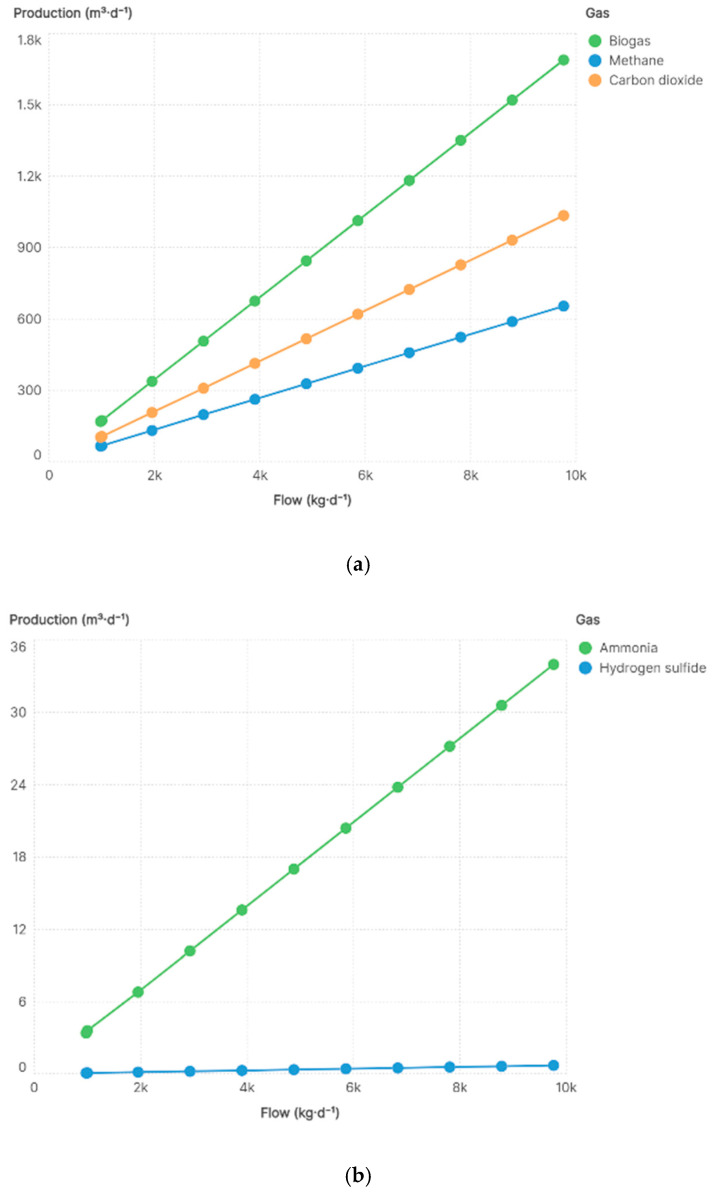
(**a**) Production of biogas, methane, and carbon dioxide; (**b**) Production of ammonia and hydrogen sulfide.

According to [36], 120–155 m^3^ of biogas can be obtained from one ton of processed filter cake, i.e., a yield of 12–15.5%. Under the base production scenario, a feed flow of 1000 kg·d^−1^ (1 t·d^−1^) of filter cake generated 172.98 m^3^·d^−1^ of biogas, corresponding to a yield of 17.2%. The simulated biogas consisted mainly of methane and carbon dioxide, while ammonia and hydrogen sulfide were produced in lower proportions (Figure 3a,b).

This value represents a theoretical upper-bound estimate derived from stoichiometric modeling and may slightly exceed experimental yields reported in the literature (12–15.5%) due to the absence of biological conversion constraints and operational inefficiencies in the simulation framework.

#### 3.4.2. Production Flow Case in Pastaza

Figure 3a shows that biogas production increased as the feed flow increased, with the gas mixture mainly composed of CH_4_ and CO_2_. Figure 3b indicates the presence of NH_3_ and SH_4_ in lower proportions. The average methane content obtained was 39.6%, while carbon dioxide reached 58.3%. Ammonia represented 2.03% of the gas mixture, whereas hydrogen sulfide remained at a low level of 0.04%.

#### 3.4.3. Boundary Conditions for the Proposed Model

**Figure 4:**
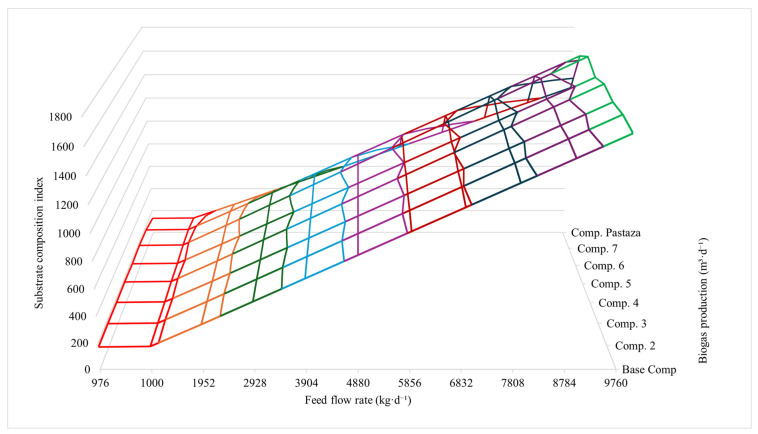
Biogas production in Pastaza. Base comp. = Base composition, Comp. = composition.

Figure 4 shows the relationship between filter cake composition, feed flow, and biogas production. Biogas output varied according to both composition and production flow. Among the evaluated scenarios, Composition 6 achieved the highest biogas production, reaching 1736.40 m^3^·d^−1^ at a feed flow of 9760 kg·d^−1^. An overall increase in biogas production was observed as the feed flow increased, particularly for Compositions 6, 7, and 8.

**Figure 5:**
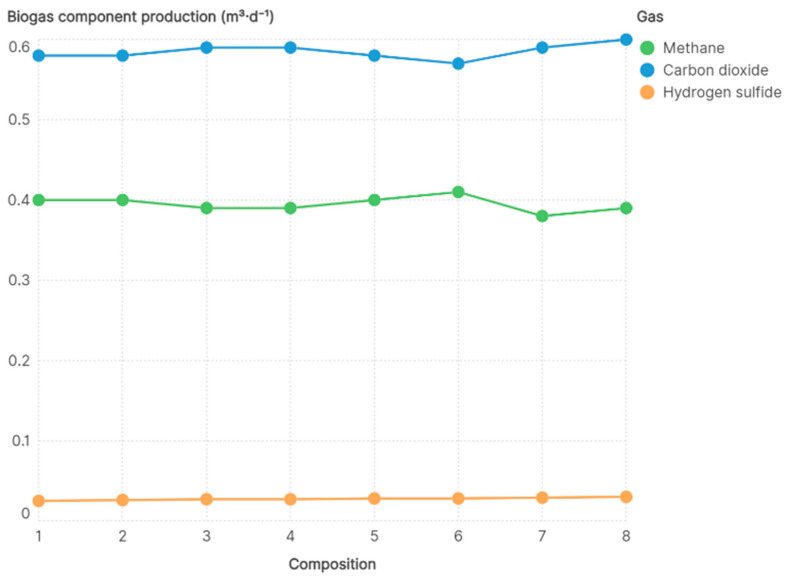
Biogas composition.

Hydrogen sulfide remained stable and below 0.12% in all formulations, as shown in Figure 5. This indicates a low concentration of sulfur compounds in the gas phase and a consistent behavior of H_2_S across the evaluated conditions.

### 3.5. Technical Evaluation of Biogas

The biogas obtained from filter cake showed an average calorific value of 13,900 kJ·m^−3^. In the highest production scenario evaluated, with a biogas flow of 1288.62 m^3^·day^−1^, an estimated saving of 1242.88 kg·day^−1^ of wood was obtained, equivalent to 1.24 t·day^−1^ of solid biomass. In Composition 6, with a methane content of 40.69%, biogas production values of 71.16 and 1736.4 m^3^·day^−1^ were obtained at filter cake feed flows of 400 and 9760 kg·day^−1^, respectively. These production levels corresponded to wood savings of 70.86 and 1728.99 kg·day^−1^, as well as bagasse savings of 135.91 and 3316.43 kg·day^−1^ (Table 6).

### 3.6. Comparison of the Environmental Impact of Gaseous Fuels Using TRACI

The environmental impact assessment using TRACI factors compared four gaseous fuels: biogas, methane (natural gas), LPG, and propane, considering the stages from production to energy use. Biogas was produced through anaerobic digestion of filter cake under mesophilic conditions and purified using iron (III) oxide, resulting in a gas with a methane content of 40.7% (Table 7). Methane, LPG, and propane were considered based on their conventional industrial production routes from fossil resources.

The results show differences among fuels in the impact categories of climate change, acidification, and photochemical ozone formation. Biogas presented the highest climate change impact, reaching 17.40 kg CO_2_ eq·m^−3^. Methane exhibited a lower value of 2.875 kg CO_2_ eq·m^−3^, while LPG and propane showed intermediate values of 3.07 and 3.05 kg CO_2_ eq·m^−3^, respectively.

In terms of acidification, biogas reached 0.00516 kg SO_2_ eq·m^−3^, followed by LPG (0.00280 kg SO_2_ eq·m^−3^), propane (0.00210 kg SO_2_ eq·m^−3^), and methane (0.00140 kg SO_2_ eq·m^−3^). For photochemical ozone formation, LPG and propane presented the highest impacts, with values of 0.09919 and 0.07440 kg O_3_ eq·m^−3^, respectively, while biogas and methane reached 0.05894 and 0.04965 kg O_3_ eq·m^−3^ (Table 7).

In addition to volumetric comparison, environmental impacts were normalized per unit of useful energy (MJ), using the lower heating value of each fuel (Table 8). This energy-based normalization enables a consistent comparison of environmental performance among fuels delivering different calorific outputs and avoids interpretations based solely on gas volume.

### 3.7. Occupational Assessment and Risk Analysis for Biogas Production

The results of the OHSP indicator calculation are summarized in Table 9. The stages of receiving and grinding the filter cake and cleaning and discharging the digestate showed the highest OHSP values, reaching 0.08 and 0.096, respectively. These stages also recorded higher numbers of accidents and lost workdays, with two accidents per year and three lost days in both cases.

Lower OHSP values were obtained for loading the biodigester (0.075) and operating the biodigester (0.048). These stages involved fewer accidents and were characterized by more controlled working conditions, with reduced direct exposure to waste and gaseous emissions. The numerical values for hours worked, accidents, days lost, and OHSP indicators for each process stage are presented in Table 9.

Operational mitigation actions compatible with artisanal facilities include partial enclosure of reception areas, assisted natural ventilation, basic mechanical aids for material handling, preventive signage, and the systematic use of personal protective equipment. These measures reduce exposure to physical, chemical, and biological hazards while maintaining low implementation complexity and gradual economic feasibility in rural contexts.

## 4. Discussion

The diversity of compounds identified in filter cake indicates the complexity of this by-product and its potential for agro-industrial use, including structural fiber, phytosterols, and residual sugars. This variability depends on crop management, sugarcane variety, and technological practices applied during processing [6].

The waste contains a wide variety of organic compounds, including polysaccharides and oligosaccharides, amino acids, long-chain fatty acids, alcohols, and phytosterols. These components determine the structure of the stoichiometric equations and lead mainly to the formation of CH_4_ and CO_2_ as final products, in agreement with previously reported mass balance-based models for anaerobic digestion [43].

The simulated configuration enables the optimization of substrate proportions and operational conditions to enhance methane production during anaerobic digestion [44]. Mesophilic operation supports process stability and biogas yield by favoring the activity of anaerobic microorganisms [28]. The separation of digestate and biogas streams improves operational control and allows independent handling of solid and gaseous products.

The incorporation of iron (III) oxide in the purification stage allows hydrogen sulfide to be converted into stable sulfide compounds such as Fe_2_S_3_, reducing corrosive effects and improving biogas quality [45]. This mechanism aligns with adsorption-based purification strategies described for biogas treatment systems [46], ensuring safer handling and greater suitability of the biogas for energy applications.

The use of variable feedstock flows allows the assessment of system behavior under realistic production fluctuations commonly observed in artisanal sugar mills. The application of a ±10% variation in filter cake availability provides a basis for identifying break-even conditions and the most viable production scenarios, considering seasonal and operational variability in the region [47]. This approach improves the robustness of the simulation by accounting for uncertainties in raw material supply.

The biogas yield obtained in this study is higher than the range of 120–155 m^3^·t^−1^, equivalent to 12–15.5%, reported for filter cake digestion in previous studies [36]. This difference can be explained by variations in substrate composition and process assumptions applied during simulation. The chemical characteristics of filter cake, influenced by agroecological conditions, crop type, operational efficiency of the sugar mill, and clarification method, play a determining role in biogas production potential [35]. These factors contribute to the variability observed among reported yields and support the use of simulation approaches to evaluate optimized operating scenarios.

Although the simulated biogas yield reaches 17.2%, this value remains below the theoretical biochemical methane potential typically reported for lignocellulosic substrates, indicating that structural constraints and conversion losses persist. Ref. [48] reported that substrate composition and recalcitrant fractions strongly limit achievable methane yields even under standardized digestion conditions. Therefore, the simulated system represents an intermediate conversion scenario rather than approaching the absolute theoretical limit.

The methane fraction obtained is slightly below the typical range of 40–70% reported for biogas systems [49] but close to the value of 42.47% reported for filter cake digestion [50]. The relatively high CO_2_ content is interpreted based on the compositional balance and stoichiometric pathways implemented in the simulation model. This association represents an inference derived from theoretical mass balance behavior rather than from direct experimental gas composition measurements, and therefore should be understood as a model-based interpretation [51]. The detection of NH_3_ indicates protein and amino acid degradation during digestion, while the very low SH_4_ concentration is consistent with efficient sulfur removal using metal oxides such as Fe_2_O_3_, which can remove 70–90% of H_2_S [52].

Higher biogas volumes do not necessarily translate into proportional increases in methane concentration because gas composition depends on the balance between methane-forming and CO_2_-generating pathways during anaerobic conversion. Substrate fractions rich in lipids and sterols favor routes that increase CO_2_ generation, affecting gas quality. Ref. [53] reported that substrate biochemical composition influences the CH_4_/CO_2_ ratio in anaerobic digestion systems. In addition, fixed stoichiometric conversion ratios constrain compositional flexibility in steady-state simulations.

From an energy perspective, lower CO_2_ concentrations are associated with improved combustion performance and higher calorific values [54]. Under the simulated conditions, the CH_4_ fraction obtained from filter cake was sufficiently high and the CO_2_ content remained at moderate levels, allowing the direct use of biogas for heating, heat generation, or combined power applications without requiring an additional CO_2_ removal stage.

Uncertainty remains associated with the use of literature-derived compositional data adjusted to local conditions, as natural variability in lignocellulosic residues may occur due to differences in crop variety, processing efficiency, clarification practices and moisture content. Variations in key fractions such as structural carbohydrates, lignin and protein directly influence substrate biodegradability, gas yield and methane concentration during anaerobic digestion. Ref. [55] demonstrated that compositional shifts in fermentable and recalcitrant fractions significantly affect biochemical methane potential and conversion efficiency. Consequently, realistic deviations from average compositions may modify predicted biogas volumes and methane quality, particularly when lignin content increases. Therefore, the reported results represent indicative trends and comparative behavior rather than absolute performance values under field conditions.

The higher biogas production observed in Compositions 6, 7, and 8 is influenced by differences in raw material composition. For instance, Composition 8 included a mixture with higher alcohol content, which favors microbial activity during the acidogenic phase and contributes to increased biogas volumes. However, despite the volumetric increase, Figure 5 shows a slight decrease in the molar fraction of methane, while carbon dioxide remained the dominant component, reaching 57.2% in Composition 6. This behavior is associated with the anaerobic degradation of phytosterols and long-chain alcohols, metabolic pathways that generate a higher proportion of CO_2_ relative to CH_4_ [56].

The superior biogas production observed for Composition 6 is primarily associated with its higher proportion of readily biodegradable fractions relative to structural and recalcitrant components. Increased availability of fermentable carbohydrates and protein enhances substrate accessibility for methanogenic conversion, while a comparatively lower contribution of lignin limits structural resistance to biodegradation. Ref. [55] shown that substrates enriched in easily degradable organic fractions exhibit higher methane conversion efficiency and faster stabilization behavior. This compositional balance explains the consistently higher gas yields obtained for Composition 6 compared with the other formulations.

No experimental measurements from local artisanal panela facilities were available for direct validation of the simulation results at the time of this study. Consequently, the model outputs should be interpreted as scenario-based estimates intended to support comparative assessment and preliminary feasibility analysis rather than predictive operational forecasting. Future experimental monitoring would allow refinement of compositional inputs and calibration of conversion assumptions.

Although present at low concentrations, hydrogen sulfide affects biogas quality due to its corrosive nature. Its limited presence may be associated with the reduced degradation of sulfur-containing amino acids such as cysteine and methionine in the protein fraction of the substrate. Effective H_2_S control in anaerobic processes can be achieved through redox condition adjustment and the use of absorbent agents, improving system stability and reducing operational risks [57].

The calorific value obtained for biogas produced from filter cake supports its technical viability as an alternative energy source for panela production systems. Its energy performance is comparable to that of traditional solid fuels commonly used in sugar mills, such as bagasse and *Eucalyptus globulus* wood, whose heating values have been widely reported in the literature [32,33]. Based on this energy equivalence, the use of 1 m^3^ of biogas is equivalent to replacing approximately 0.96 kg of wood or 1.85–1.91 kg of bagasse, depending on biogas composition (Table 6).

Comparable resource-oriented assessments have been reported for residual biomasses such as beach-cast macroalgae, where high organic carbon content and favorable biochemical composition enable biofuel and biogas generation pathways. Ref. [58], demonstrated that macroalgal residues exhibit significant potential for bioenergy conversion when appropriate preprocessing and valorization strategies are applied. Although the substrate characteristics differ, both systems share the strategic advantage of transforming locally accumulated organic residues into renewable energy carriers, supporting regional sustainability frameworks.

When extrapolated to continuous annual operation, the estimated biomass savings reach 631.08 t·year^−1^, corresponding to the conservation of 3.63 ha·year^−1^ of Amazon rainforest or approximately 902 trees·year^−1^. This ecological equivalence was established using reference values for aboveground biomass density and average individual tree biomass reported for Amazonian forests [34], providing an environmental interpretation of the energy substitution achieved.

The magnitude of biomass displacement obtained in this study exceeds that reported for domestic biodigester systems, where average savings of 1856.78 kg of wood·year^−1^ per installed unit have been documented, equivalent to 0.011 ha·year^−1^ of preserved forest [59]. This difference is primarily associated with the operational scale of the system, as household biodigesters are designed to supply individual families, whereas the present evaluation considers higher volumes of filter cake generated in artisanal sugar mills.

The estimated wood savings are also consistent with findings from high-altitude rural communities, where biogas systems with methane contents ranging from 35 to 60% have been shown to replace up to 75% of daily firewood consumption, with reported savings of 0.1–0.4 kg per person per day, equivalent to approximately 1 m^3^ of biogas·person^−1^·day^−1^ [60]. This consistency supports the robustness of the simulated results under different geographic and socio-productive contexts.

The equivalence between biomass savings and preserved forest area represents a simplified indicator intended to communicate potential biomass displacement effects. The calculation assumes generic wood supply characteristics and does not explicitly differentiate between primary forest extraction, secondary regrowth or managed plantation sources. Ref. [61] highlighted that carbon stock density and biomass productivity vary substantially across forest types, influencing the interpretation of land-use equivalence indicators. Therefore, the reported conservation estimates should be interpreted as indicative approximations rather than precise land-use substitution metrics.

In addition to energy substitution, replacing the direct combustion of wood and bagasse with biogas contributes to a reduction in emissions of particulate matter, carbon monoxide, and volatile organic compounds, thereby decreasing exposure to pollutants in artisanal heating environments [62]. Furthermore, the diversion of bagasse traditionally used as fuel enables its use as a low-impact agro-industrial input for applications such as composting, artisanal paper production, and lignocellulosic board manufacturing, reinforcing circular resource use and reducing pressure on forest biomass [63,64].

The higher climate change impact observed for biogas is associated with its lower methane concentration and the presence of unburned CH_4_ and biogenic CO_2_ in its composition. Methane leakage during production and handling plays a critical role, as leakage rates exceeding 2% can offset the climate benefits of biogas when compared with natural gas [65].

The environmental assessment considers only gaseous emissions, and therefore excludes digestate handling, storage and downstream utilization. This boundary definition may underestimate or bias certain impact categories, particularly those associated with nutrient release, land application and secondary emissions. Ref. [66] reported that digestate management pathways can significantly influence life-cycle environmental performance in anaerobic digestion systems. Consequently, the present results should be interpreted within this system boundary constraint, and future assessments may benefit from integrating alternative digestate utilization scenarios.

Natural gas shows a lower climate impact due to its high purity, with methane concentrations close to 98%, which favors more efficient combustion and lower emissions per unit of useful energy delivered [67]. In contrast, LPG and propane exhibit intermediate climate impacts, reflecting their fossil origin and combustion characteristics.

The acidification potential of biogas is mainly related to the presence of hydrogen sulfide. During anaerobic digestion, a significant fraction of sulfur contained in the substrate can be converted into H_2_S, which, if not adequately removed, dominates the acidification profile of biogas systems [68]. This highlights the importance of effective gas purification prior to utilization.

The higher contribution of LPG and propane to photochemical ozone formation is linked to emissions of nitrogen oxides and volatile organic compounds during combustion and handling. These emissions favor tropospheric ozone formation and explain the higher smog-related impacts observed for these fuels [69]. Overall, the comparison shows that although biogas presents higher impacts in certain categories, its renewable origin and potential for emission mitigation through improved control strategies support its role as a sustainable alternative to fossil gaseous fuels.

The comparatively higher climate change impact observed for biogas is primarily associated with methane leakage sensitivity and reduced combustion efficiency linked to lower CH_4_ purity, while biogenic CO_2_ is typically considered climate-neutral. Small leakage rates may disproportionately increase greenhouse gas impact due to methane’s high global warming potential. Ref. [70] reported that improvements in gas upgrading efficiency, leakage control and combustion optimization substantially reduce climate impacts of biogas systems and may enable performance comparable to or better than fossil fuels. Therefore, operational improvements represent critical leverage points for climate competitiveness.

The higher OHSP values observed during filter cake reception and digestate cleaning are associated with increased exposure to physical, chemical, and biological hazards, including wet waste handling, hydrogen sulfide and ammonia emissions, noise, and elevated temperatures. These stages represent critical points for occupational risk management in biogas production systems. Incorporating occupational indicators into S-LCA frameworks allows environmental performance to be linked with worker health and safety, identifying biomass handling and waste gas management as priority stages [71].

Risk reduction strategies applicable to artisanal systems include partial mechanization of material handling, enclosed reception zones to reduce exposure, assisted ventilation for gas dispersion, procedural controls for cleaning operations and systematic use of personal protective equipment. Ref. [72] reported that low-cost engineering controls and organizational measures significantly reduce occupational exposure in small-scale biogas facilities without compromising operational feasibility. Linking risk identification with practical mitigation actions supports safer implementation pathways.

The identification of ATEX hazardous areas is essential for reducing accident risks related to methane and hydrogen sulfide leaks or accumulations in biogas facilities [73]. In this context, the digestate cleaning and discharge stage presents elevated risk due to the simultaneous presence of toxic vapors and thermal stress. Effective risk reduction requires integrated preventive strategies that combine regulatory compliance, organizational measures, and worker training, supported by appropriate personal protective equipment, adequate ventilation, and clear safety signage [74]. The use of respirators with acid gas filters, waterproof gloves, and insulated protective clothing contributes to minimizing exposure and improving occupational safety during high-risk operations.

Integration of energy recovery, environmental performance and occupational risk highlights relevant trade-offs across sustainability dimensions. Scenarios with higher biogas generation improve biomass displacement and energy autonomy but may increase exposure intensity and sensitivity to methane leakage. Conversely, conservative operational configurations reduce occupational risk and environmental sensitivity while limiting achievable energy recovery [75]. These interactions underline the need for balanced system optimization rather than single-criterion maximization.

From an economic perspective, the utilization of locally generated organic residues for bioenergy production has been associated with reduced feedstock acquisition costs, lower waste management expenses and opportunities for decentralized energy supply in regional production systems. Ref. [19], reported that residual biomass valorization pathways, including biogas generation, may support local economic viability by integrating waste recovery, energy substitution and by-product utilization within circular resource chains. In the context of artisanal panela production, the on-site availability of filter cake and the partial substitution of conventional fuels by biogas, as observed in the simulated scenarios, may contribute to improved resource efficiency and local energy autonomy.

Despite the technical potential identified, real-world implementation in artisanal panela facilities may face barriers related to initial investment costs, operational complexity, maintenance capacity and user training requirements. Small-scale producers often operate under limited financial margins and technical support availability, which may constrain technology uptake [76]. Addressing these barriers requires simplified system design, modular deployment strategies and capacity-building initiatives to support long-term operational stability.

## 5. Conclusions

The energy use of filter cake through anaerobic digestion is a viable option for the artisanal sugarcane industry in the province of Pastaza. Composition 6 performed best in the process, with biogas production of up to 1736.40 m^3^·day^−1^ and methane fractions close to 40.7%. This level enables the partial replacement of wood and bagasse used as fuels and is associated with an approximate annual reduction of 631.08 t·year^−1^ of solid biomass, equivalent to the conservation of around 3.63 ha·year^−1^ of Amazon rainforest or about 902 trees per year. The environmental analysis showed that the sustainable performance of the system depends on controlling methane leaks and removing H_2_S to reduce contributions to climate change and acidification, while the social assessment identified greater occupational risks in the stages of receiving the filter cake and cleaning the digestate.

## Figures and Tables

**Table 1:** Boundary conditions for composition.

	Lower Limit	Upper Limit	Base Comp.	Comp. 2	Comp. 3	Comp. 4	Comp. 5	Comp. 6	Comp. 7
Moisture			70.72	70.72	70.72	70.72	70.72	70.72	70.72
Protein	3.81	4.68	AVG Protein	Lower limit Protein	Upper limit Protein	AVG Protein	AVG Protein	AVG Protein	AVG Protein
Lipids	3.22	4.1	AVG Lipids	Upper limit Lipids	Lower limit Lipids	AVG Lipids	AVG Lipids	AVG Lipids	AVG Lipids
Ash	2.34	3.51	AVG Ash	AVG Ash	AVG Ash	Lower limit Ash	Upper limit Ash	AVG Ash	AVG Ash
Sucrose	2.93	4.1	AVG Sucrose	AVG Sucrose	AVG Sucrose	Upper limit Sucrose	Lower limit Sucrose	AVG Sucrose	AVG Sucrose
Fiber	4.98	7.32	AVG Fiber	AVG Fiber	AVG Fiber	AVG Fiber	AVG Fiber	Lower limit Fiber	Upper limit Fiber
Others	7.61	9.96	AVG Others	AVG Others	AVG Others	AVG Others	AVG Others	Upper limit Others	Lower limit Others

AVG = Average value, Base comp. = Base composition, Comp. = composition.

**Table 2:** Boundary conditions for feedstock flows.

	Flow for Pastaza
F (100%)	THV
F (90%)	THV-(90% * THV)
F (80%)	THV-(80% * THV)
F (70%)	THV-(70% * THV)
F (60%)	THV-(60% * THV)
F (50%)	THV-(50% * THV)
F (40%)	THV-(40% * THV)
F (30%)	THV-(30% * THV)
F (20%)	THV-(20% * THV)
F (10%)	THV-(10% * THV)

* THV = theoretical value, F = flow.

**Table 3:** Detailed composition of the filter cake.

Amino Acids/(%)	Value	Author	Fatty Acids/(%)	Value	Author	Minerals and Others/(%)	Value	Author
Crude protein	4.39	[25]	Total lipids	3.51	[27]	Ash	2.93	[25]
Aspartic acid	0.56		Myristic acid	0.013		Silicon (Si)	0.189	[26]
Threonine	0.33		Palmitic acid	0.385		Zinc (Zn)	0.357	
Glutamic acid	0.47		Stearic acid	0.082		Iron (Fe)	1.401	
Methionine	0.06		Oleic acid	0.084		Magnesium (Mg)	0.49	
Isoleucine	0.27		Linoleic acid	0.025		Copper (Cu)	0.028	
Alanine	0.74		Linolenic acid	0.04		Manganese (Mn)	0.112	
Valine	0.45		Arachidonic acid	0.004		Aluminum (Al)	0.35	
Leucine	0.46		n-tetracosanoic acid	0.013		Sucrose	3.52	[25]
Tyrosine	0.08		n-hexacosanoic acid	0.006		Fiber	6.15	
Phenylalanine	0.17		n-octacosanoic acid	0.488		Cellulose	4.378	[5]
Tryptophan	0.15		n-nonacosanoic acid	0.023		Hemicellulose	1.181	
Histidine	0.28		n-triacontanoic acid	0.286		Lignin	0.59	
Lysine	0.27		n-dotriacontanoic acid	0.158		Others	8.78	[25]
Arginine	0.12		n-tetratriacontanoic acid	0.212		n-tetracosanol	0.712	[27]
			Stigmasterol	0.524		n-hexacosanol	0.558	
			Campesterol	0.572		n-heptacosanol	0.294	
			β-sitosterol	0.602		n-octacosanol	6.086	
						n-nonacosanol	0.294	
						n-triacontanol	0.872	
						n-dotriacontanol	0.172	
						n-tetratricontanol	0.335	

**Table 4:** Organic compounds and corresponding chemical reactions.

Component	Chemical Reaction
Oligosaccharide	
Sucrose	$C_{12}H_{22}O_{11}+H_{2}O\to 6CH_{4}+6CO_{2}$
Polysaccharides	
Hemicellulose	$C_{421}H_{810}O_{405}+16H_{2}O\to 210.5CH_{4}+210.5CO_{2}$
Cellulose	$C_{240}H_{400}O_{200}+40H_{2}O\to 120CH_{4}+120CO_{2}$
Cross-linked phenolic polymers	
Lignin	$C_{9.94}H_{11.72}O_{4.17}+4.93H_{2}O\to 5.39CH_{4}+4.55CO_{2}$
Amino acids	
Aspartic acid	$C_{4}H_{7}O_{4}N+H_{2}O\to 1.5CH_{4}+NH_{3}+2.5CO_{2}$
Fatty acids	
Linoleic acid	$C_{18}H_{32}O_{2}+9H_{2}O\to 12.5CH_{4}+5.5CO_{2}$
Higher primary aliphatic alcohols	
N-tetracosanol	$C_{24}H_{50}O+11H_{2}O\to 18CH_{4}+6CO_{2}$
Phytosterols or sterols of plant origin	
Stigmasterol	$C_{29}H_{48}O+16.5H_{2}O\to 20.5CH_{4}+8.75CO_{2}$

**Table 5:** Production flows in Pastaza.

Percentage of Total Flow Fed	Flow for Pastaza (kg·d^−1^)
10%	976
20%	1952
30%	2928
40%	3904
50%	4880
60%	5856
70%	6832
80%	7808
90%	8784
100%	9760

**Table 6:** Results of estimated fuel savings.

Feed Flow (kg·d^−1^)	Biogas Production (m^3^·d^−1^)	Bagasse Saved (kg·d^−1^)	Wood Saved (kg·d^−1^)
1	1	1.909	0.996
400	71.16	135.91	70.86
9760	1736.4	3316.43	1728.99
Annual ecological equivalent		631.08 t·year^−1^ (≈3.63 ha ≈902 trees)	

**Table 7:** Assessment of the environmental performance of gaseous fuels using TRACI factors.

Fuel	Climate Change (kg CO_2_ eq)	Acidification (kg SO_2_ eq)	Smog (kg O_3_ eq)
Biogas	17.40	0.00516	0.05894
Methane (natural gas)	2.875	0.00140	0.04965
LPG	3.07	0.00280	0.09919
Propane	3.05	0.00210	0.07440

**Table 8:** Environmental impact normalized per unit of useful energy.

Fuel	Climate Change (kg CO_2_ eq/MJ)	Acidification (kg SO_2_ eq/MJ)	Smog (kg O_3_ eq/MJ)
Biogas	1.252	0.000371	0.00424
Methane (natural gas)	0.080	0.000039	0.00139
LPG	0.033	0.000030	0.00107
Propane	0.033	0.000023	0.00080

**Table 9:** Occupational risk analysis in biogas production using the OHSP indicator.

Process Stage	Main Risks	Hours Worked/Year	Accidents/Year
Receiving and grinding the filter cake	Noise, dust, and heat	1800	2
Loading the biodigester	Contact with waste and gases	1600	1
Operating the biodigester	Internal pressure and methane gas	2000	1
Cleaning and discharging the digestate	Biological and thermal risk	1500	2

## Data Availability

All the data generated in the research is in the manuscript.
